# Effect of wavefront optimized LASIK on higher order aberrations in myopic patients

**DOI:** 10.12669/pjms.315.7683

**Published:** 2015

**Authors:** Muhammad Saim Khan, Sadia Humayun, Aisha Fawad, Mazhar Ishaq, Sabahat Arzoo, Fawad Mashhadi

**Affiliations:** 1 Dr. Muhammad Saim Khan, MBBS. Armed Forces Institute of Ophthalmology, Mall Road, Rawalpindi, Pakistan; 2 Dr. Sadia Humayun, MBBS, MCPS, FCPS, FRCS. Armed Forces Institute of Ophthalmology, Mall Road, Rawalpindi, Pakistan; 3 Dr. Aisha Fawad, MBBS, FCPS. Armed Forces Institute of Ophthalmology, Mall Road, Rawalpindi, Pakistan; 4 Prof. Dr. Mazhar Ishaq, FRC Opth, FRCS, FCPS. Armed Forces Institute of Ophthalmology, Mall Road, Rawalpindi, Pakistan; 5 Sabahat Arzoo, BSc (Hons) in Optometry and Orthoptics. Armed Forces Institute of Ophthalmology, Mall Road, Rawalpindi, Pakistan; 6 Dr. Fawad Mashhadi, MBBS, MPH, MCPS, MPhil. Armed Forces Institute of Ophthalmology, Mall Road, Rawalpindi, Pakistan

**Keywords:** Coma, Spherical Aberration, Higher order aberrations, LASIK

## Abstract

**Objective:**

To determine mean change induced in root mean square value of higher order aberrations in myopic patients undergoing wavefront optimized laser assisted in situ keratomileusis.

**Methods:**

This quasi experimental study was conducted at Armed Forces Institute of Ophthalmology, Rawalpindi, Pakistan from Jan 2014 to Dec 2014. Sixty eyes of 35 myopic patients were included in the study. All patients underwent wavefront optimized (WFO) laser assisted in situ keratomileusis (LASIK) using femtosecond laser (FM 200Wavelight technologies) and excimer laser (Ew 500Wavelight technologies). Higher order aberrations (HOAs) were measured with aberrometer (Wavelight allegro analyzer version 1073) during preoperative assessment and one month after surgery.

**Results:**

All 35 patients ranged from 20 to 32 years with a mean age of 24 ± 3.41 years. Refractive error ranged from -1.00 to -9.50 DS with a mean spherical equivalent (SE) of -3.73 ± 1.95 before surgery and - 0.36 ± 1.50DS one month after LASIK. Uncorrected visual acuity (UCVA) was improved to 0.00 or better in all 60 eyes. An increase of 1.56 fold was observed in RMS of total HOAs. Among the HOAs, a statistically significant positive correlation was observed between spherical aberrations (4^th^ order aberration) and preoperative spherical equivalent.

**Conclusion:**

In spite of excellent improvement in refractive error, significant amount of higher order aberrations were induced after WFO LASIK.

## INTRODUCTION

LASIK is the most popular and commonly performed procedure in the field of corneal refractive surgery.^1^ Laser refractive surgery works on the principle of modification of corneal refractive power by means of photo ablation of the stromal tissue.^2^ Earlier surgical procedures like Radial keratotomy, Arcuate keratotomy and Photorefractive keratectomy proved to be successful in quantitative improvement of refractive error, however it was noticed that they degrade the quality of vision by reducing night vision clarity, glare, halos.^3^ Studies have shown that higher order aberrations (HOAs) are responsible for these post-operative visual complaints.^3^,^4^ The introduction of wave front technology has brought revolution in the field of corneal refractive surgery because of better predictability and increased accuracy. The reliable results of wavefront guided refractive surgical procedures has not only resulted in increasing the number of patients being benefit but also the expansion of surgical indications.^1^,^4^,^5^

Higher order aberrations (spherical aberrations, coma, trefoil) are small optical irregularities of the ocular refractive media. Unlike low order aberrations (myopia, hypermetropia, simple astigmatism) they cannot be corrected with spectacles or contact lenses.^6^ They are commonly described in terms of Zernike polynomials and measured by aberrometer which measures the root mean square (RMS) value in micrometers.^7^,^8^ Zernike polynomials are divided into several orders, low order aberrations (first and second order), and high order aberrartions (third order onwards).^7^,^8^ Studies have shown that wave front optimized LASIK, though corrects the refractive error but increases the HOAs.^9^,^10^

The rationale of conducting this study is to analyze the induced change in HOAs by wavefront optimized LASIK in myopic patients as there has been no study of this kind conducted in our population.

## METHODS

This was a Quasi experimental study conducted at Armed Forces Institute of Ophthalmology, Rawalpindi, Pakistan from Jan 2014 to Dec 2014. Sample size was calculated on the basis of Open EPI info calculator and appeared to be 55 eyes. In order to overcome the problem of loss to follow up we included 60 eyes of 35 subjects in the study by non-probability (purposive) sampling technique. Patients with age ranging from 18 – 40 years and refractive error ranging from -1.00 D to -12.00 D were included. Patients with preoperative corrected distant visual acuity (CDVA) or postoperative uncorrected visual acuity (UCVA) worse than 20/20 (log MAR value 0.00), ocular allergy, corneal ectasia, history of ocular trauma or ophthalmic surgery were excluded.

All patients underwent preoperative ophthalmic clinical examination that included manifest and cycloplegic refractions, UCVA, CDVA, slit lamp examination and measurement of RMS of HOAs with aberrometer (Wavelight allergo analyzer version 1073). In order to generate more accurate and reliable results, patients were instructed to stop using contact lenses for at least 02 weeks prior to aberrometry. WFO LASIK was performed in all the patients. Thickness of flap was kept to 110 um and created with femtosecond laser (FM 200 Wavelight technologies) while excimer laser (Ew 500Wavelight technologies) was used to ablate corneal stroma leaving behind at least 300 um residual stromal bed. Postoperative evaluation was performed 01 month after surgery and included UCVA, CDVA, manifest refraction and RMS value of HOAs using aberrometer. All wavefront measurements were repeated three times and the best image was selected.

Results of aberrometry were analyzed taking a constant pupil diameter of 6 mm. Mean RMS values of total HOAs, coma (3^rd^ order aberration) and spherical aberrations (4^th^ order aberration) were calculated. Statistical package for social sciences (SPSS 17.0) for windows was used for statistical analysis. The data was described in terms of mean ± SD (standard deviation). The induced change in HOAs comparing the preoperative and postoperative measures were evaluated statistically with sample *t*-test, paired sample test and linear regression (p ≤0.05 significance level).

## RESULTS

**Fig.1:**
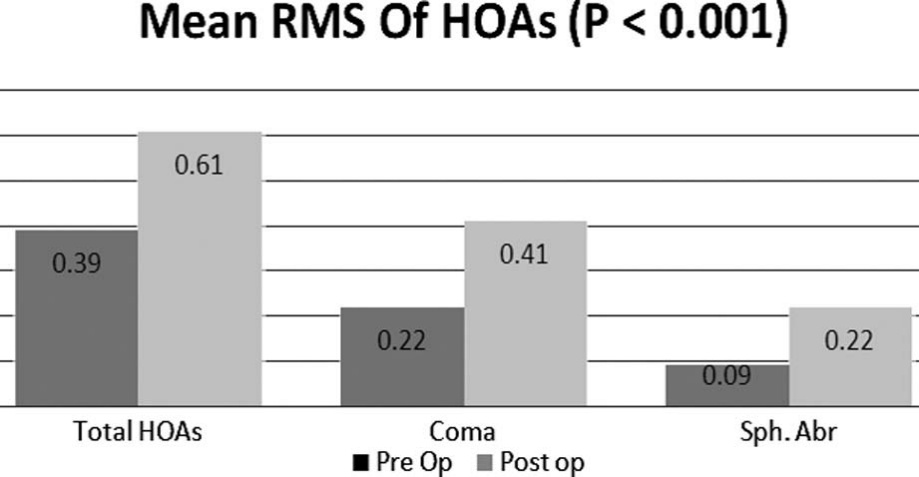
Comparison of the root mean square (RMS) of HOAs before and after LASIK.

Total 60 eyes of 35 patients (21 females and 14 males) underwent WFO LASIK. Out of total, 31 were right eyes while 29 were left eyes. Age of patients ranged from 20 to 32 years with mean age of 24 ± 3.41 years. Preoperative SE of refractive error ranged from -1.00 DS to -9.50 DS with a mean of 3.733 ±1.96 and CDVA in all patients was 0.00 or better. Postoperative SE of refractive error ranged from 0.00 DS to 0.56 DS with a mean of -0.36 ± 0.15 and UCVA in all the eyes was 0.00 or better. The mean RMS value before and after LASIK was calculated (Table-I, Fig.1). All eyes had a statistically significant change (p < 0.05) in HOAs after the procedure. (Table-II). The magnitude of induced change in coma (-0.18±0.17) was more than spherical aberrations (0.132±0.36). There was 1.56 times increase in RMS of total HOAs (Table-III) after surgery and a weak but statistically significant positive correlation between induced change in spherical aberrations and pre LASIK spherical equivalent of refractive error. (Table-IV).

**Table-I:** Difference in pre LASIK and post LASIK values of spherical equivalent (SE) and HOAs.

Variable	Pre LASIK	Post LASIK	P value
Mean SE	-3.73 ± 1.95	-0.36 ± 0.15	<0.001
Mean S. Abb	0.09 ± 0.71	0.22 ± 0.12	<0.001
Mean Coma	0.22 ± 0.17	0.41 ± 0.24	<0.001
Mean Total HOAs	0.39 ± 0.24	0.61 ± 0.33	<0.001

**Table-II:** The induced change in mean RMS and Stanadard deviation of Total HOAs, Coma and Spherical aberrations.

Variables	Induced Change	Std. Deviation	P value
Post Total HOAs - Pre Total HOAs	0.21867	0.20357	P < 0.001
Post Coma - Pre Coma	0.18600	0.17940	P < 0.001
Post.S.Abb - Pre S.Abb	0.13233	0.13694	P < 0.001

**Table-III:** No of times HOAs have changed after LASIK.

	Pre LASIK	Post LASIK	Ratio Post/Pre
Total RMS	0.39	0.61	1.56
Coma	0.22	0.41	1.86
Spherical Aberration	0.09	0.22	2.44

**Table-IV:** Correlation between attempted spherical equivalent and induced change in higher order Aberrations.

Variable	Regression Equation	r -value coefficient of correlation	p-value
Induced change in S.Abb with attempted SE	0.022-0.029	0.422	0.001
Induced change in Total HOAs with attempted SE	0.121-0.026	0.25	0.052
Induced change in Coma with attempted SE	0.129-0.015	0.168	0.20

## DISCUSSION

The popularity of laser refractive surgery has led to increase in the number of studies evaluating the visual functions and quality of vision. Most of the patients after laser refractive surgery had significant improvement in the visual acuity in terms of refractive error correction which made them get rid of glasses. However, many of them had variable complaints about visual problems e.g. glare, poor night vision and decreased contrast sensitivity.^10^-^15^

This decrease in quality of vision is linked to increase in HOAs after laser refractive surgery.^16^,^17^ Recent introduction of wavefront guided modalities like wavefront optimized and customized surgery has been claimed to improve the postoperative visual outcome after laser refractive surgery because they not only correct the refractive error but also decrease the HOAs. Like Levy et al most of authors have concluded that visual results are better after wavefornt guided laser ablations than conventional surgery.^18^-^21^ However, others could not find any significant difference between wavefront guided and conventional treatment.^22^,^23^

In our study we performed wavefront optimized LASIK, and studied the change in HOAs. We found 1.56 times increase in total HOAs and our results were better than mentioned by Padmanabhan et al.^9^ and Barriuso et al.^24^ who claimed it to be 1.96 times and 1.9 times respectively. The ratio of post LASIK total HOAs to pre LASIK total HOAs (Table-III) was more similar to that concluded by Padmanabhan et al.^9^ and Barriuso et al.^24^ than Seiler et al.^25^ Our results with coma (3^rd^ order aberrations) were better than those reported by others,^9^ however, Padmanabhan et al.^9^ and Barriuso et al.^24^ found the ratio of post LASIK RMS of spherical aberrations to pre LASIK RMS of spherical aberrations to be 3.99 and 1.86 respectively while we found it to be 2.44 times. Contrary to Seiler et al, we found largest induced change in coma. Like other authors, we found a statistically significant but weak positive correlation between the amount of spherical aberrations induced to pre LASIK refractive error (Table-IV).^24^,^25^

The currently available literature regarding the clinical importance of HOAs and the potential benefit of correcting them made us study the effect of WFO LASIK on HOAs. We believe that our results are important but studies on larger sample size are needed to further evaluate, analyze and compare not only the changes in HOAs after LASIK but also the effect of these changes on patients` quality of vision. This would lead the visual outcomes of corneal refractive surgery further step ahead by designing customized techniques and algorithms that will correct both low order and higher order aberrations.

## CONCLUSION

Inspite of remarkable improvement in refractive error, significant amount of higher order aberrations were induced after WFO LASIK.
